# Structural Complexity and Plasticity of Signaling Regulation at the Melanocortin-4 Receptor

**DOI:** 10.3390/ijms21165728

**Published:** 2020-08-10

**Authors:** Gunnar Kleinau, Nicolas A. Heyder, Ya-Xiong Tao, Patrick Scheerer

**Affiliations:** 1 Group Protein X-ray Crystallography and Signal Transduction, Institute of Medical Physics and Biophysics, Charité-Universitätsmedizin Berlin, Corporate Member of Freie Universität Berlin, Humboldt-Universität zu Berlin, and Berlin Institute of Health, D-10117 Berlin, Germany; gunnar.kleinau@charite.de (G.K.); nicolas-andreas.heyder@charite.de (N.A.H.); 2 Department of Anatomy, Physiology and Pharmacology, Auburn University College of Veterinary Medicine, Auburn, AL 36849, USA; taoyaxi@auburn.edu; 3 German Centre for Cardiovascular Research (DZHK), Partner Site Berlin, D-10785 Berlin, Germany

**Keywords:** G protein-coupled receptor, melanocortin receptors, melanocortin-4 receptor, signal transduction

## Abstract

The melanocortin-4 receptor (MC4R) is a class A G protein-coupled receptor (GPCR), essential for regulation of appetite and metabolism. Pathogenic inactivating *MC4R* mutations are the most frequent cause of monogenic obesity, a growing medical and socioeconomic problem worldwide. The MC4R mediates either ligand-independent or ligand-dependent signaling. Agonists such as α-melanocyte-stimulating hormone (α-MSH) induce anorexigenic effects, in contrast to the endogenous inverse agonist agouti-related peptide (AgRP), which causes orexigenic effects by suppressing high basal signaling activity. Agonist action triggers the binding of different subtypes of G proteins and arrestins, leading to concomitant induction of diverse intracellular signaling cascades. An increasing number of experimental studies have unraveled molecular properties and mechanisms of MC4R signal transduction related to physiological and pathophysiological aspects. In addition, the MC4R crystal structure was recently determined at 2.75 Å resolution in an inactive state bound with a peptide antagonist. Underpinned by structural homology models of MC4R complexes simulating a presumably active-state conformation compared to the structure of the inactive state, we here briefly summarize the current understanding and key players involved in the MC4R switching process between different activity states. Finally, these perspectives highlight the complexity and plasticity in MC4R signaling regulation and identify gaps in our current knowledge.

## 1. The Melanocortin-4 Receptor

The G protein-coupled receptor (GPCR)-linked modular and plastic signaling system is one of the main signaling pathways in eukaryotes and evolved early in eukaryotic evolution [1,2]. These membrane-spanning receptors and respective interaction partners are associated with most physiological processes, such as regulation of tissue development, body growth, memory development, reproduction, behavior, or senses such as taste or vision [3]. The relevance of approximately 830 human GPCRs [4] is based on their proven or, in the case of the still orphan receptors, suspected key roles in signal transduction from the extracellular side to the intracellular interior induced by a huge variety of stimuli [5]. Receptor stimulants (agonists) can be, e.g., odors, diverse metabolites, ions, mechanical forces, peptides, nucleotides, light, amines, or even large glycoproteins [6]. Receptor stimulation by agonistic ligands results in the stabilization of an active-state conformation with an increased capacity for intracellular coupling and activation of, e.g., G proteins or arrestins [7]. GPCR/G protein interaction triggers activation of second messenger signaling pathways regulating downstream events such as ion channel activity or gene expression [8,9].

Malfunctions of approximately 100 GPCRs are associated with different human diseases [10], including viral infections, cancer, infertility, inflammation, as well as metabolic and neurological disorders [11,12,13,14,15,16]. The pathogenic triggers at GPCRs vary from hormonal dysregulation, virus infections, or interactions with auto-antibodies, as well as mutations. This fact, along with their key role in cell signaling, explains why approximately ~35% of FDA-approved drugs target GPCRs [17].

GPCRs share a common structural architecture consisting of an extracellular N-terminus (Nt), an intracellular C-terminus (Ct), seven transmembrane helices (TMHs) connected via three intracellular (ILs), and three extracellular loops (ELs). Currently, over 300 GPCR structures from more than 70 unique receptors were determined by protein X-ray crystallography or by cryo-electron microscopy (cryo-EM), including different conformations or substates (inactive and active states) and receptor complexes with G proteins, arrestins, or peptides derived from both, respectively [18,19,20]. Such structural information, although far from being complete for the full set of GPCR families and classes, is an indispensable source of information for scientific studies related to cell physiology, molecular biology, pharmacology, and medicine [7,20].

A milestone in melanocortin-4 receptor (MC4R) research was the recently published crystal structure in an inactive state, with a resolution of 2.75 Å [21]. This 3D structure provides insights into the general structure of the inactive MC4R, as well as binding details of an antagonistic peptide ligand (SHU9119 [22]), which is supposedly mediated or supported by calcium ions. The MC4R belongs to class A GPCRs and is one of five melanocortin receptor subtypes (MC1-5R) [23,24,25]. It is involved in regulating various physiological processes, including energy homeostasis, cardiovascular function, glucose/lipid homeostasis, and sexual functions [25,26]. MC4R activation results in decreased food intake and increased energy expenditure, hence decreased body weight [27]. In the cardiovascular system, MC4R activation increases heart rate and blood pressure, whereas MC4R deficiency causes dilated cardiomyopathy in mice [28]. MC4R also promotes glucose homeostasis, independent of its action on the body weight, providing additional therapeutic benefit (reviewed in [25]). The involvement of MC4R in regulating reproduction has resulted in an FDA-approved drug for treating hypoactive sexual desire disorder in women [29]. More recently, MC4R has been shown to be involved in regulating glucagon-like peptide-1 and peptide YY secretion from enteroendocrine L cells [30], modulating serum insulin levels [31], and liver and limb regeneration [32,33].

Agonist action (e.g., by α-melanocyte-stimulating hormone (α-MSH)) in the MC4R induces an appetite-reducing effect [34], in contrast to the inverse agonist ligand agouti-related peptide (AgRP) with appetite-enhancing effect [35,36]. This endogenous counter-regulation mechanism at the ligand level is of specific interest, since other endogenous inverse agonists have not been described for GPCRs so far, with the exception of agouti-signaling protein for the MC1R [24] and the inverse agonistic molecule retinal permanently bound in rhodopsin [37]. An important interrelated MC4R characteristic is relatively high constitutive (basal) Gs-mediated adenylate cyclase activation [38], which can be increased further by agonist binding. Moreover, other signaling pathways associated with Gq, Gi, or arrestin activation by MC4R are also known [25,39]. Importantly, several external factors are involved in the signaling regulation of the MC4R and were identified in recent years, such as homo- and hetero-oligomerization [40,41], the interacting melanocortin-2 receptor accessory protein 2 (MRAP2) [42,43,44], or the endogenous ligand lipocalin-2 produced by osteoblast in bone [45].

To date, *MC4R* gene mutations inactivating the MC4R for signaling (loss of function) are the most frequent monogenic cause of obesity [46]. This is also of importance because obesity is related to different comorbidities, such as type 2 diabetes mellitus or cardiovascular disease [47]. To date, approximately 165 missense mutations in 129 different residues have been reported [48]. Several of these pathogenic MC4R mutations have been observed to cause biased signaling through the MC4R by forcing preference for a specific signaling pathway [39,49,50,51,52] (reviewed in [35]). Moreover, a newly developed MC4R ligand causing biased signaling, setmelanotide, was applied successfully in anti-obesity treatment [39]. While a huge amount of specific synthetic or peptidic ligands, including setmelanotide [53,54,55,56], bivalent ligands [57,58,59,60], and many others [61,62,63], have been designed, there is a tremendous medical as well as socioeconomic interest in the development of functionally optimized (tailor-made for specific signaling pathways) MC4R ligands reasoned by an increasing prevalence of human obesity worldwide [64].

**Figure 1:**
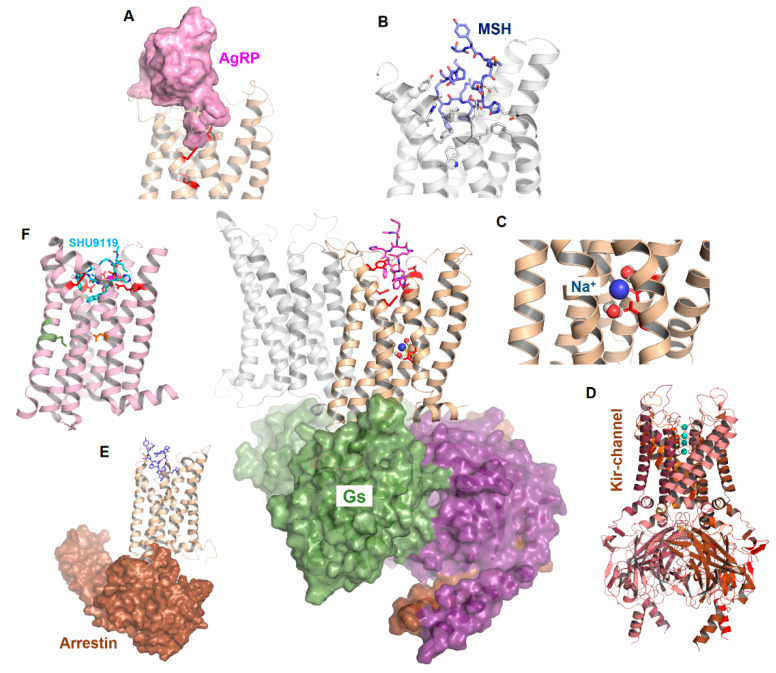
Scheme of MC4R interrelations. The figure reflects functional-structural aspects related to MC4R signal transduction. Middle: Potential interaction of an MC4R dimer with a heterotrimeric G protein (surface representation). (**A**) represents the binding of AgRP, (**B**) the binding of α-MSH, and (**C**) the potential binding of sodium. (**D**) indicates an association with other proteins such as Kir channels and (**E**) arrestin binding with the MC4R. (**F**) The recently described crystal structure of the MC4R in the inactive state is bound to a peptide antagonist (SHU9119).

In this review, we briefly describe current knowledge on the structural properties and involved factors related to MC4R signal transduction. This summary is supported by structural models of active-state ligand/MC4R/effector complexes (Figure 1) in comparison to recent insights from the inactive MC4R/antagonist complex structure [21]. We aimed to highlight this receptor as part of a molecular machinery where several intrinsic and external factors regulate signaling.

## 2. Specific Features in the MC4R Sequence and Structure Linked with Functionalities

In addition to the above mentioned common structural GPCR features, such as seven TMHs and their connecting loops, which are confirmed by the recently solved inactive-state structure of the MC4R [21], the MC4R is also characterized by amino acids in particular sequence regions that are responsible for specific peculiarities in the protein structure. In the following sections, we will discuss and highlight structural features related to functional properties, and will also specify open questions and current gaps in knowledge.

### 2.1. A Presumed Role of the MC4R N-terminus in Regulating Permanent Basal Activity and the Inhibitory Effect of AgRP

**Figure 2:**
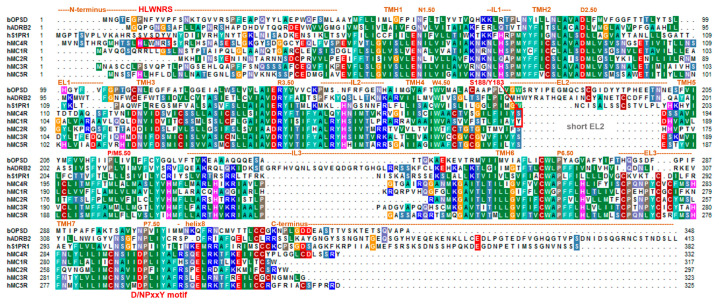
Sequence alignment of the MC4R and homologous MCRs in comparison to bovine rhodopsin (bOPSD), the β_2_-adrenergic receptor (ADRB2), and the sphingosine 1-phosphate receptor 1 (S1PR1). This comparison visualizes predicted structural dimensions of each MC4R part, also assigned according to the recently solved MC4R inactive-state structure (PDB ID: 6W25) [21], such as the helices or loops (IL, intracellular loops; ELs, extracellular loops; TMH, transmembrane helices). Highly conserved positions, according to the unifying Ballesteros and Weinstein numbering scheme for class A GPCRs [83], are indicated by respective numbers. This alignment was visualized using the BioEdit software [84]. Specific background colors indicating conservation (Blossum62 matrix) and reflecting biophysical properties of the amino acid side chains as follows: black—proline, blue—positively charged, cyan/green—aromatic and hydrophobic, green—hydrophobic, red—negatively charged, gray—hydrophilic, dark-red—cysteine, and magenta—histidine.

Previous studies [65] suggested that the MC4R is one of the few known GPCRs [66,67] where the N-terminus has a fundamental impact on signaling regulation by acting as an intramolecular (tethered/bound) ligand. The general mechanism of a tethered ligand is suspected not only for the MC4R but also for GPR83 [68], glycoprotein hormone receptors (GPHRs) [69,70,71], S1P2R [72], LGR7 [73], α_1D_-adrenoceptor [74], adhesion GPCRs (aGPCR) [75], or protease-activated receptors (PARs) [76]. Tethered ligands can permanently activate the receptor to a certain degree compared to full ligand-stimulated activity, as suggested for the MC4R, or they can be silent in the basal state and be activated by enzymatic clipping, as shown for the PARs [76]. Of note, for GPHRs, it was proposed that intramolecular inverse agonism mediated by the N-terminus on the transmembrane domain [77] can be interconverted by TSH binding into intramolecular agonism [70], or that a specific extracellular region acts as a tethered ligand that becomes activated by hormone-ligand binding [69]. Moreover, a recent study on GPR52 showed that its EL2 occupies the orthosteric binding pocket, acting as a “built-in agonist”, conferring an intrinsically high basal activity to this receptor [78]. Such a scenario for basal activity regulation is excluded for the MC4R, reasoned by a very short EL2 structure (see section below, Figure 2 and inactive-state MC4R crystal structure [21]).

**Figure 3:**
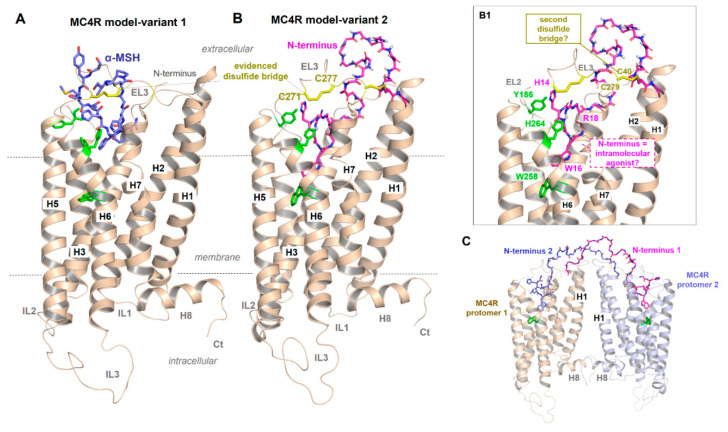
Presumed MC4R active-state models. (**A**) Independent of various details, this receptor shows structural features that are common for GPCRs such as seven transmembrane helices (H1–7) and corresponding connection loops (extracellular loops, EL1-3, and intracellular loops, IL1-3), the additional membrane surface helix H8 as well as the N-terminus (Nt) and C-terminus (Ct). As recently proposed [39], α-MSH may bind into a crevice between EL1, EL2 and EL3 and transitions to the transmembrane helices. A disulfide bridge (yellow sticks) in the EL3 has also been proposed for the MC2R [85]. (**B**) The second MC4R model variant is intended to show how parts of the N-terminus (magenta, sticks) might interact as intramolecular ligands, which should lead to a permanent basal signaling activity as previously proposed [65,79]. Details of this interaction [79] are shown in (**B1**) (labeled side chains, green for MC4R, magenta for the N-terminus). In addition, a second previously proposed disulfide bridge in the extracellular region should be located between the N-terminus (C40) and the EL3 (C279), whereby alanine mutations of these two cysteines have no impact on MC4R functionalities [86]. In the solved inactive-state crystal structure of MC4R [21], no disulfide bridge between C40 and C279 can be observed, only between C271 and C277 in EL3. (**C**) It would also be hypothetically conceivable that in an MC4R homodimer (see oligomerization section below), the N-termini between the two individual monomers transinteract from one to the other, respectively, in contrast to a monomeric receptor constellation (*cis* activation (**B1**)).

For the MC4R, a specific part of the MC4R N-terminus was proposed to be embedded between the TMHs and ELs, acting as an intramolecular tethered (partial) agonist on the MC4R by permitting high basal signaling activity [65,79]. The minimal “activating ^14^HLWNRS^19^ sequence” (human MC4R motif sequence (Figure 2); underlined are the most mutation-sensitive amino acids) is supposed to interact intramolecularly with the amino acid residues of the TM helical bundle (e.g., F201, W258, and F262) [79] (Figure 3). Notwithstanding, conservation of the MC4R N-termini between positions 14 and 19 (human MC4R numbering) among orthologous receptors is high; however, several significant variations can be observed in different animal species, such as birds (motif H**F**W**MH**S), fish (**Y**/**FR**N**H**S) or reptiles (H/**X**F/**X**WNHS/**T**) (bold letters indicate residues deviating from the human sequence, see also Supplemental material in [48]). Assuming a conserved mechanism of signaling regulation in vertebrates and, therefore, also for the presumed relationship between this sequence motif and maintenance of the MC4R basal activity, the question arises of how such deviations may impact basal receptor activity of diverse animal branches and its impact on AgRP action. Indeed, we showed recently that fish MC4Rs have several-fold higher basal activities than human MC4R, when assayed in the same experiments [26,80,81,82].

Inverse agonist activity of AgRP on the MC4R was suggested to be attributed to the inhibition of the N-terminus-mediated permanent constitutive activation of the receptor [79]. How exactly AgRP interacts or competes with an already bound N-terminus and/or simultaneously interacts with the TMH bundle, as previously presumed [87,88], and how α-MSH binds into a region that partially overlaps with the putative binding site for the N-terminus (Figure 3B,B1) is unclear but can be definitively answered by a full-length MC4R structure bound with AgRP or α-MSH.

Importantly, the amino acid sequence in the recently published MC4R structure [21] was modified to improve protein stability to obtain sufficient amounts of protein in a crystallizable conformation (introduction of thermostabilizing mutations, the N-terminus of the receptor truncated from amino acid positions 1 to 15, and insertion of a fusion protein into ICL3). Unfortunately, this resulted in a structure in which insights into the potential contribution of the core unit (residues 14–19) as part of a potential intramolecular ligand [65,79] are lacking. Moreover, of the five MCR subtypes, only MC4R exposes such an “activating ^14^HLWNRS^19^” N-terminal sequence motif (Figure 2), which would exclude a similar mechanism for other MCR subtypes, yet they share endogenous ligands, such as α-MSH or AgRP (MC3R).

Another assumption should be that in case of an MC4R homodimer arrangement [40,41,89,90], the N-terminus between the two monomers of the homodimer should cross-interact (Figure 3C). Interestingly, transactivation has indeed been shown for the MC4R [91], while a contribution of the N-terminus to this mechanism has not yet been studied. Finally, in a recent protein docking study based on advanced molecular dynamics simulations on the MC4R and a small antagonistic molecule [92], the N-terminus of the MC4R was predicted to contribute to a multistep binding mechanism. However, despite several indications of a potential impact of the extracellular N-terminus on MC4R signal transduction, the definitive role of this part is not yet fully understood and requires further experimental studies for clarification.

### 2.2. Basal (Constitutive, Ligand-Independent) Signaling Activity

In terms of the proposed role of the N-terminus in regulating basal activity, an important question to be raised here concerns the current state of information available on the regulation of this feature for the MC4R. Principally, high basal activity has a physiological significance not only for the MC4R [25] but also for other GPCRs, e.g., the opioid receptors [93]. Basal activity of GPCRs can be particularly modified by several factors, such as the pH, receptor variants, the level of receptor cell surface expression, or interaction with specific ions [94,95]. As described above, for the MC4R, increased basal activity has been suggested by the action of an intramolecular agonist [65,79] but recently, an alternative mechanism of basal activity regulation in the MC4R by interaction with divalent ions was published [96]. In particular, zinc ions cause activation of the cAMP pathway in the MC4R, whereby copper reduces MC4R activity to below normal basal signaling levels [96]. Hence, the authors concluded that zinc causes a certain level of permanent MC4R signaling activity as an external factor, which would be a prerequisite for an inverse agonistic effect of AgRP. The effect of zinc ions as agonists (positive allosteric modulators) in the MC4R has been previously reported [97]. However, it remains unclear how this finding on basal activity regulation of the MC4R by ions correlates or interferes with the presumed intramolecular agonistic binding and effect of the N-terminus [65,79]. It is also possible that zinc ion binding to the MC4R releases constraining interactions that maintain the MC4R in inactive conformation to increase basal signaling. Further studies on cation regulation of MC4R might be very fertile.

The MC4R has been shown to interact with both MRAP1 and MRAP2 [43,44,98], and this interaction can affect MC4R expression on the cell surface, ligand binding, and basal and agonist-stimulated signaling, with the exact effect different in different species (reviewed in [42,99]). For example, MRAP1 enhances the maturation of human MC4R [100]. The interaction between MRAP2 and the MC4R as a heteromer has a functional effect on ligand binding preferences, with the MC4R becoming highly sensitive to adrenocorticotropin (ACTH) binding, which favors a direct structural interaction between the two proteins [44] and a mutual impact on their functionalities. MRAP1 also increase the basal activity of human MC4R [101]. In other animals, different functional effects are observed. For example, in a teleost, MRAP2 co-expression leads to decreased basal and agonist-stimulated Gs-cAMP signaling but increased basal ERK1/2 signaling [81]. There are further known examples of GPCRs that are functionally and structurally associated with non-GPCR proteins, with some serving as receptor-specific molecular chaperones [102,103], such as the calcitonin gene-related peptide (CGRP) receptor, which is a heterodimer of the calcitonin receptor-like receptor (CLR) class B GPCR [104] and a type 1 transmembrane domain protein, the receptor activity-modifying protein 1 (RAMP1) [105]. RAMPs play an essential role in the presentation of CLR on the cell surface. In addition, RAMPs, which have a structured N-terminal extracellular domain (ECD) with approximately 100 amino acids, a single transmembrane domain and a short intracellular C-terminus, can allosterically influence the signaling activity of class B GPCRs [106,107]. The question to be addressed here is whether the RAMP-class B-GPCR interaction mechanism is somehow similar to that of MRAP2–MC4R.

Basal activity is an important pharmacological property of a particular GPCR; either increased (constitutively active mutant) or decreased basal activity can cause human diseases [108]. In general, the occurrence or non-occurrence of basal activity of a particular GPCR is likely associated with the linked biological background, particularly in terms of a permanent physiological function, as is, for example, known for the mammalian thyrotropin receptor vs. the homologous follitropin receptor (time/development-dependent activity) [109]. Basal activity may facilitate further activation by the ligand, e.g., an increased affinity for the agonist and lowering the intramolecular energy barrier to overcome the inactive receptor state towards receptor activation [94]. For example, lutropin receptor has higher basal activity and is more prone to mutation-induced constitutive activation than follitropin receptor [110]. Of note, for viral chemokine receptors, a more active basal state is associated with promiscuous G protein signaling through a broader set of activated G protein subtypes compared to human receptor orthologues [111]. For the MC4R, the basal activity is related to the fine-tuned up and down signal regulation by the agonist α-MSH and the inverse agonist AgRP, respectively. Dysfunctional basal signaling has been identified in naturally occurring mutations in the MC4R identified from obese patients [65]. It is important to note that AgRP is described as a biased ligand, which reduces basal Gs activation of the MC4R but also activates ERK1/2-mediated signaling [35,112,113,114]. This indicates a very specific role of basal activity in this receptor–ligand–effector constellation by combining a physiological relevance of permanent signaling activity (anorexigenic effect) with distinct effector activation mechanisms for anorexigenic effects by AgRP [38].

Another important aspect is that constitutive MC4R activity inhibits L-type voltage-gated calcium channels (CaV), specifically subtypes CaV1.2/1.3 and CaV2.1 [115]. Additionally, agonist-dependent inhibition of CaV2.2 and basal cAMP signaling MC4R activity was shown to differentially impact these CaV subtypes through Gs and Gi/o pathways [115]. Remarkably, the inactive MC4R, regarding its inability to induce cAMP generation (via Gs activation), is proposed to couple and activate the ion channel Kir7.1 [116]. This indicates a G protein (Gs)-independent mechanism of the MC4R to regulate ion channel activity [21], presumably by direct interaction between MC4R and Kir channels. Such heteromer interactions are known for GPCRs and ion channels (e.g., [117]), or GPCRs and functionally related 12-helix substrate transporters [118]. These interactions should enable a rapid and direct mutual transfer of information between functionally related proteins of different superfamilies. Given that the MC4R is known to activate different intracellular signaling pathways depending on ligands [35,39,49,51,113], future studies should investigate in detail how MC4R functionalities (signaling pathways and ligand binding properties) are modulated by accessory proteins, such as ion channels or MRAPs [42,43,44].

### 2.3. The Short Second Extracellular Loop Has A Strong Impact on MC4R Functional Properties

**Figure 4:**
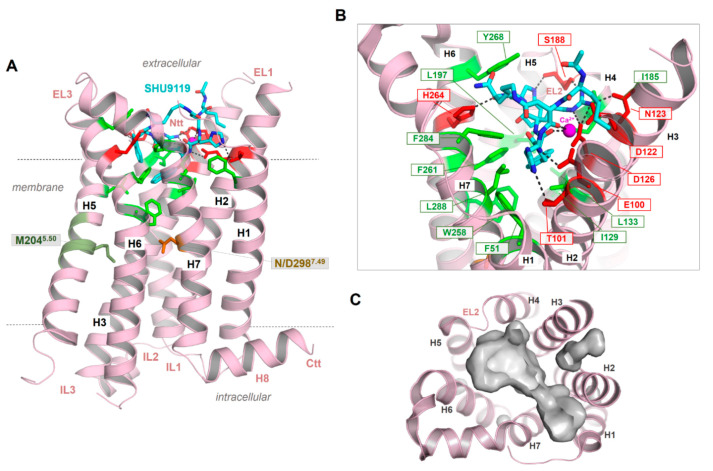
The crystal structure of the MC4R in an inactive state bound with the antagonist SHU9119 [21]. (**A**) The structure shows a number of important details of the MC4R, such as an unkinked helix 5. Instead of a highly conserved proline in class A GPCRs, a methionine is located at the corresponding position 5.50 (deep green). The NP^7.50^xxY motif in helix 7 (the numbers 5.50 and 7.50 are used according to the unifying numbering scheme for amino acids in the transmembrane helices of class A GPCRs proposed by Ballesteros and Weinstein [83]), which is highly conserved in class A GPCR, is a less conserved DPxxY motif in the MCR subtypes. However, in the crystallized MC4R structure, D7.49 has been mutated to an asparagine (NP^7.50^xxY) motif. The peptidic antagonist SHU9119 (cyan) binds between the EL2 and several helix-loop transitions. (**B**) SHU9119 binding is characterized by hydrogen bonds (lack dotted lines) to hydrophilic amino acid residues (red sticks) and backbone atoms in TMH 2–4 and the EL2. Further participating residues in TMH6 and TMH7 are hydrophobic and aromatic (green sticks). Experimental studies combined with electron densities (resolution of the crystal structure is 2.75 Å) between D122, D126, and E100 indicating calcium binding in this region, which is also associated with ligand binding. (**C**) The empty ligand binding pocket (inner surface, gray) reflects the dimension of this crevice, which is large in the MCRs compared to other GPCRs due to the very short EL2.

In contrast to most of the other class A GPCRs, the MC4R EL2 has been reported to only consist of three to four amino acids (e.g., [48]), which has been recently confirmed by the inactive MC4R crystal structure [21] (Figure 2 and Figure 4). Usually, in class A GPCRs, the EL2 comprises ~10–30 amino acids and is located centrally above the transmembrane helical bundle. The EL2 in class A and class B GPCRs is important for ligand binding and signaling regulation [119,120,121,122,123,124,125]. A highly conserved disulfide bridge among different classes of GPCRs between the EL2 and TMH3 (conservation of a cysteine in TMH3 at position 3.25 in class A GPCRs is ~88%) is an essential structural feature and constrains the EL2 in its spatial allocation embedded between EL1 and EL3. However, in addition to the MC4R without cysteines in EL2 and TMH3, there are only a few other GPCRs without a disulfide bridge between EL2 and TMH3, e.g., S1P1R, GPR82, GPR139, or GPR142 and GPR146. While many mutagenesis studies on these particular cysteines in EL2 and/or TMH3 of GPCRs result in non-functional (not expressed on the cell surface) proteins (e.g., [126,127]), it is remarkable that all MCR subtypes and the other examples mentioned above do not require such a structural feature.

As a result of the very short EL2 in the MC4R and other MCR subtypes, the inner volume of a crevice between the ELs and transitions to the helices is broader compared to GPCRs with a longer EL2. This crevice (Figure 4C) is known as the preferred ligand binding region in class A GPCRs, at least also for allosteric modulators (e.g., [128]). The enlarged extracellular crevice of the MCRs should open the possibility for interactions with large hormone-ligands, such as AgRP or bind the N-terminus intramolecularly, as previously suggested [65,79]. In addition, the recently published MC4R crystal structure in an inactive state [21] revealed that a peptide ligand and divalent calcium ion could be bound simultaneously in this extracellular cavity (Figure 4), which is so far unique for GPCRs.

Another interesting feature observed in the new MC4R crystal structure is the direct interaction between SHU9119 and S188 in the EL2 through a hydrogen bond [21]. In agreement with other GPCR examples, this supports a fundamental role of the EL2 for MC4R function, in particular for peptide ligand binding. It remains unknown whether this interaction between the EL2 and the antagonist SHU9119 is also relevant for agonistic ligand binding; however, it should be important for the distinction between agonistic and antagonistic ligand action. Similarly, atomic details of binding of other small-molecule agonists (such as THIQ, [129]) and antagonists (such as ML00253764, [130]) to the MC4R remain to be elucidated. Of special interest is the observation of orthosteric vs. allosteric binding of these small-molecule ligands to MC4R of different species [26,114,131]; the molecular basis of this differential binding and signaling effect remains to be investigated.

### 2.4. Structural Specificities of the MCRs in the Transmembrane Region

Class A GPCRs share conservation of specific amino acids or amino acid motifs in the transmembrane region [83,132,133], which are required for correct protein folding, stability, and function. They participate in signal transduction as “micro-switches” (amino acid residues, such as D2.50, R3.50, W6.48), or motifs (“micro-domains”), such as NP^7.50^xxY, CWxP^6.50^, E(D)R^3.50^Y or Y^5.58^(x)7K(R)^5.66^) [134,135]. The micro-switch residues are interrelated with each other in a network of interactions, also under the participation of water molecules and ions [136,137,138].

In addition to the above described missing EL2–TMH3 disulfide bridge, the MCRs are further characterized by two deviations from these common class A GPCR amino acids. First, a proline (P5.50) is highly conserved in class A GPCRs (~80%) and supports a kink and bulge within helix 5. Proline distorts the regular helix geometry due to steric conflicts with the preceding residue and the loss of internal backbone hydrogen bond typical in a α-helix. These proline-generated kinks can be functionally important because they create structural weaknesses, thereby facilitating helix-associated movements required for interaction with intracellular partners [139,140]. As a result, most TMH5 (and other TMHs with a proline) in class A GPCR are not regularly (straight) formed, and amino acid side chains are slightly rotated around the helical axis [141]. In the MC4R, a methionine (human MC4R Met204 (Figure 4)) instead of a proline at position 5.50 causes a more regular α-helix conformation of TMH5, as is now confirmed by the recently published inactive-state MC4R structure [21]. Further examples of GPCR crystal structures without proline in TMH5 are, for instance, the S1PR1 (PDB ID: 3V2W [142]), the P2Y12 receptor (PDB ID: 4NTJ [143]), the cannabinoid receptors (PDB IDs: 5ZTY, 5TGZ [144,145]), or the lysophosphatidic acid receptor 1 (LPAR1, PDB ID: 4Z34 [146]). A regular and non-kinked conformation of TMH5 should be of particular importance, because the orientation of certain amino acid side chains in the TMH5 region to the transmembrane core depends on this structural property and, therefore, they participate in the ligand binding region of the MC4R.

**Figure 5:**
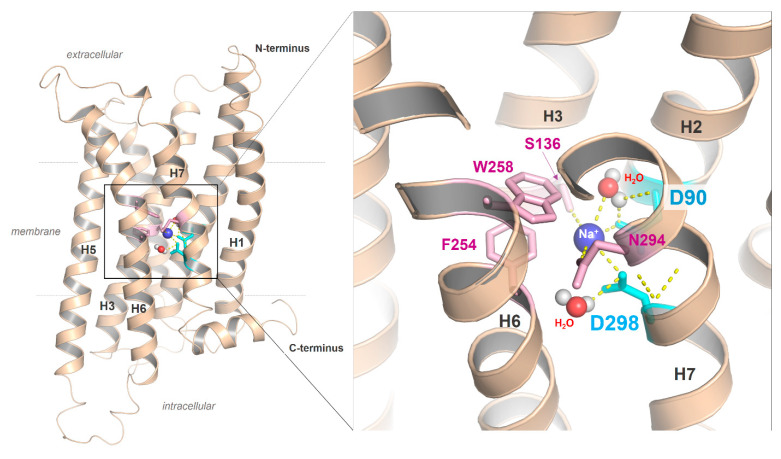
The putative sodium binding site with two aspartates in the MC4R. Sequence specificity of MCRs is a negatively charged aspartate in the DPxxY motif of transmembrane helix (H) seven, whereas in most class A GPCRs, an asparagine (NPxxY) is usually present. The MC4R aspartate in TMH7 could affect the ion binding properties of the MC4R, including the H-bond network with water molecules or the exact spatial location of the cation. In addition to the two aspartates located in TMH2 and TMH7, further amino acids, such as those from helix 3 (e.g., S136), are involved in the formation of the sodium binding site.

Second, the highly conserved class A GPCR amino acid motif **N**P^7.50^xxY in TMH7 is a **D**PxxY motif in the MC4R (Figure 2). Of note, almost 20% of class A GPCRs, with the exception of olfactory class A GPCRs, are characterized by an aspartate at position 7.49 instead of the more conserved asparagine; however, it remains unclear what structural or functional consequences are associated with this deviation [136]. Based on mutagenesis and signaling studies (e.g., [147]) or previously solved receptor structures [148], the amino acid at position 7.49 (Asn or Asp) was deliberated as contributing to the binding of positively charged sodium ions in an interaction network between water molecules and the highly conserved negatively charged aspartate at position 2.50 in TMH2 (Figure 5). An interaction with a monovalent sodium cation should be a common mechanism of allosteric modulation of GPCRs, which appears to be specifically associated with the maintenance of an inactive state (e.g., [148,149,150,151]), the orientation of TMH7 and receptor dynamics [138], implying regulation of transitions between different receptor activity states [136,148]. Interestingly, in active-state GPCR structures, no sodium ions have been observed at this site. Sodium ion exchange with the cytosol could be a key step in the activation of several GPCRs [152].

Of note, in the inactive-state MC4R structure [21], the endogenous aspartate at position 7.49 was mutated with asparagine to achieve higher thermostability (Figure 4). Therefore, this structure cannot be used to gain deeper insights into the endogenous sodium binding site of MCRs linked with the DP^7.50^xxY motif.

### 2.5. Ion Binding Sites in the MC4R

The negatively charged aspartate at position 7.49 in the MC4R described above (Figure 2 and Figure 5), in contrast to an uncharged asparagine, should lead to a modified sodium binding site in terms of binding capacity and cation affinity [136]. For the hMC4R, previous mutagenesis studies showed that the D298A mutation (D7.49A) does not significantly affect ligand binding but has impaired signaling capacity [153]. However, it was shown that there is no difference in the WT and D7.49N mutant in either ligand binding (to α-MSH or AgRP) or signaling (basal or ligand-stimulated) [154]. The substitution of D7.49N and an additional four other amino acid substitutions and C- and N-terminal truncations in the MC4R construct used for crystallization leads to a significant increase in melting temperature [21], suggesting that D7.49N might have some structural effect. Of note, a double aspartate constellation (D2.50/D7.49), as observed in MCRs and, e.g., in the P2Y12 [155], was suggested to potentially bind divalent cations [136], which is an interesting note with respect to previous reports on the impact of calcium [156,157] and a recent publication on zinc and copper binding in the MC4R [96]. Both zinc and copper ions in the presence of calcium are presumed to be negative allosteric modulators of ligand binding; however, calcium is required for high-affinity ligand binding.

This suggestion is substantiated by the MC4R crystal structure complex and accompanying experiments [21], which revealed the direct involvement of a calcium ion in SHU9119 binding at the extracellular binding crevice (Figure 4). Of note, direct structural detection of calcium ions in the extracellular region by, e.g., single-wavelength X-ray diffraction (SAD) phase determination on protein crystals with a resolution higher than 2.75 Å, will be required [21]. Native SAD determination depends crucially on the accurate detection of the weak anomalous scattering signal of light atoms, such as calcium and magnesium present in the crystallized sample [158]. Consequently, any divalent cation could be principally bound at the supposed extracellular calcium binding site but also a highly coordinated water molecule. However, in addition to previously reported calcium binding at the MC4R [156,157], radio-ligand binding experiments with ^125^I-NDP-MSH and competitive binding experiments with α-MSH confirmed the effects of Ca^2+^ on agonist binding to the MC4R. In contrast, calcium has no impact on AgRP binding. Further evidence for the supposed calcium binding was provided by investigations of the thermal stability of the MC4R as a complex with various MC4R ligands, such as SHU9119, NDP-MSH, α-MSH, and AgRP, in response to Ca^2+^, Mg^2+^, and Zn^2+^ ions [21]. Only Ca^2+^ ions increase the thermal stability of MC4R-SHU9119 or MC4R-NDP-MSH.

**Figure 6:**
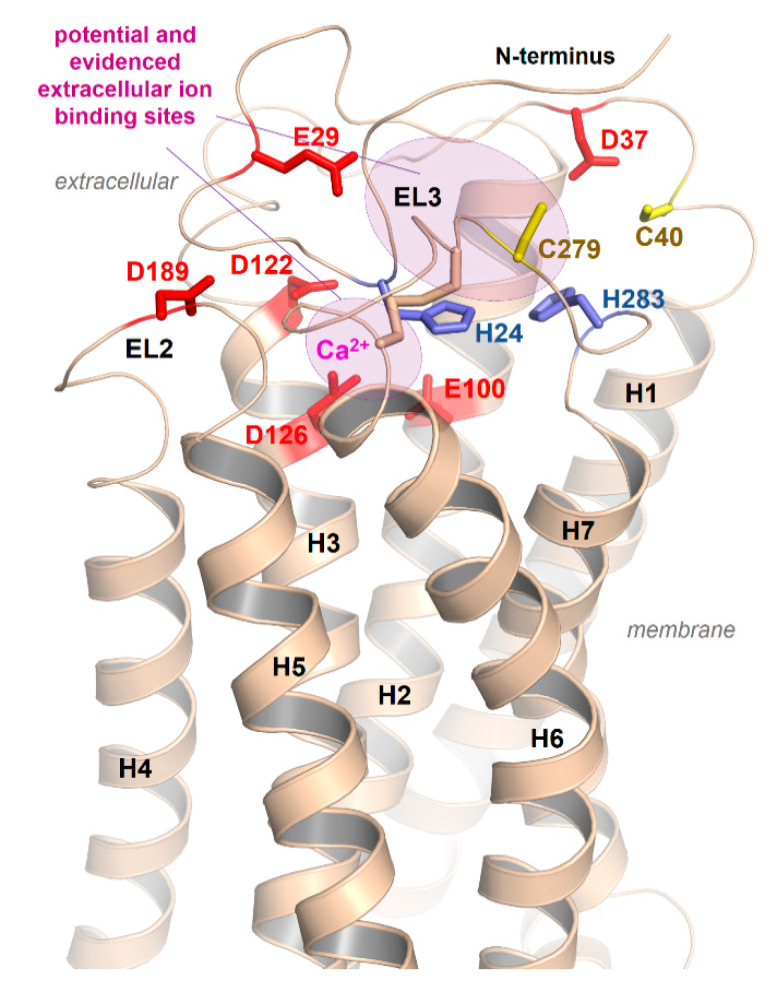
Potential extracellular cation binding sites at the MC4R. In addition to the above-described sodium binding sites between TMH2, -3, and -7 (Figure 5) and the detected calcium binding site close to the extracellular part (Figure 4) [21], several additional extracellular amino acids (negatively charged as Asp and Glu, or His and Cys as zinc binding motifs) should contribute to copper, calcium, or zinc ion binding as indicated in the model.

Our homology model of an active MC4R conformation (Figure 6) suggests that parallel to E100 (TMH2), D122, and D126 (TMH3), which were presumed by the recent crystal structure to interact with calcium [21], several other residues, such as aspartates, histidines, and cysteines in the N-terminus or the ELs, should be arranged in specific clusters capable of interacting with positively charged cations (Figure 6).

Regarding the binding of different ions to GPCRs, it was recently shown that sodium ions enhance an inactive adenosine 2A receptor (A_2A_R) conformation [159], thereby retaining an “ionic lock”, while divalent cations, such as Ca^2+^ and Mg^2+^, shift the equilibrium towards an active-state conformation. In the same study, positive allosteric effects of divalent cations were shown to be more pronounced with agonist and a G protein-derived peptide. A strong impact of Mg^2+^ ion on receptor functionalities was also found for the oxytocin receptor (OXTR), whereby Mg^2+^ ion impedes ligand binding [160]. Moreover, in addition to Ca^2+^ binding in the calcium-sensing receptor (CaSR, class C GPCRs) with multiple ion binding sites [161], structurally significant calcium binding was also found in the N-terminal domain (subdomain “low-density lipoprotein” of the leucine-rich repeat containing GPCR 7 (LGR7 or relaxin binding receptor 1 (RXFP1)) [162].

In summary, ion binding in GPCRs generally appears to be of crucial importance for the stabilization of certain conformations and activity states. Furthermore, the MC4R interacts with various ions, such as Na^+^, Ca^2+^, Zn^2+^, or Cu^2+^, and such interactions are likely to be involved in regulating basal signaling activity, ligand binding, and activity state switching processes. A deeper understanding of these interactions will be important in the future from a structural as well as physiological and pharmacological perspective.

### 2.6. Peptidic Ligand Binding at the MC4R

Of the five MCRs, MC2R is only activated by ACTH, whereas the other four MCRs can be activated by both α- and β-MSHs, and γ-MSH preferentially binds to the MC3R [25,163,164,165,166]. Of the two endogenous antagonists, agouti antagonizes MC1R, although when expressed ubiquitously, it can also antagonize MC4R [167]. AgRP is an antagonist for the MC3R and MC4R [168]. MSH binding induces receptor structure modifications, thereby enabling intracellular recruitment of arrestins and G proteins such as Gs, Gi/o, Gq/11, and G12/13 [39,169,170], which subsequently leads to stimulation or inhibition of intracellular effectors, e.g., cAMP accumulation [171] or extracellular regulated kinases 1/2 (ERK1/2) [113,169,172]. Although AgRP is an inverse agonist at the canonical Gs-cAMP pathway [173,174], it can serve as an agonist in other signaling pathways, including activating Gi/o-protein [169], arrestin-mediated internalization [175], coupling to potassium channel Kir7.1 [116], and activation of ERK1/2 [112,113,114]. The structural basis of this biased signaling is presently not understood.

Synthetic cyclic MSH derivatives [22,176], such as setmelanotide [53,54,55,56] or bremelanotide (also termed PT-141, Vyleesi, Rekynda; a cyclic heptapeptide derivative of MSH [29] that has been approved for the treatment of female hypoactive sexual desire disorder [29,177,178,179]), and multivalent [59,60] as well as non-peptidic MC4R ligands [63,180], were developed for pharmacological treatment options using the MC4R as a target. Notably, different MC4R ligands may be associated with differentially initiated signaling pathways, as already shown for setmelanotide, which varies from α-MSH in its signaling profile [39]. This observation requires common and unique mechanisms associated with MC4R ligand binding and the associated set of signaling pathways. Unfortunately, most studies on MC4R ligands, including bivalent ligands, lack comprehensive data on signal profiling, making it difficult to gain deeper insight into their differentiation at the molecular and structural level or to accurately determine the links between in vitro and in vivo effects.

**Figure 7:**
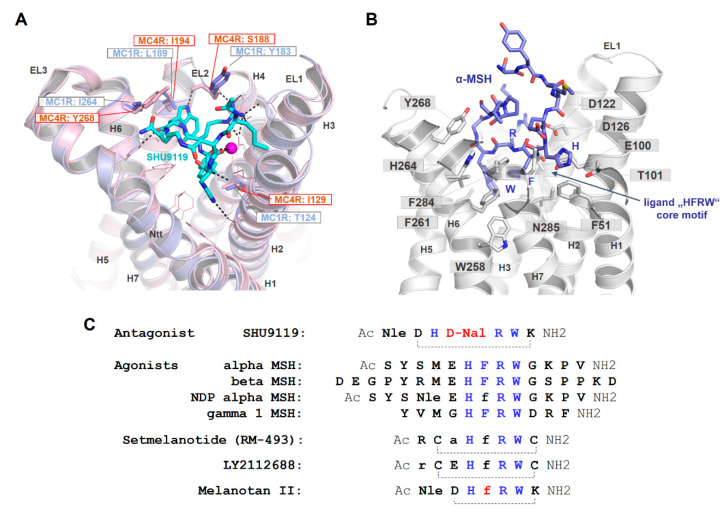
Binding site comparison between the MC4R bound with SHU9119 and the MC1R, and α-MSH in complex with the MC4R. (**A**) The MC4R (light-magenta backbone) binds SHU9119 into a cavity between the extracellular loops (ELs) and the transmembrane helices (H). Visualized are amino acids covering the ligand binding site (lines). Sticks (labeled) represent residues in the binding site that vary compared to the MC1R. These four different amino acids are also shown for the MC1R (blue) at a superimposed MC1R model based on the MC4R crystal structure. Specifically, the difference in corresponding MC4R S188 and MC1R Y183 is likely highly important for SHU9119 binding, because this ligand interacts with the side chain of S188 that should differ in the MC1R with a tyrosine at this position. In contrast to an antagonistic effect of SHU9119 on the MC4R, this ligand activates the MC1R. (**B**) Because all peptidic MCR ligands share a core motif essential for recognition by the receptor (see also C), few essential interactions observed for SHU9119 in the complex crystal structure with the MC4R may also be involved in MSH binding, e.g., (α-MSH/receptor) H6/T101 and R8/D126. Using these interactions as constraints for α-MSH docking into the MC4R, W9, in particular, may be differentially located in the receptor compared to W7 of SHU9119. This is essentially caused by the large and bulky aromatic D-Nal (D-Naphtylalanine) in SHU9119 instead of the F7 in α-MSH, which leads to differences in the orientation of the tryptophan. (**C**) This sequence comparison highlights the similarities and distinct features of MC4R ligands. Of note, the chemical difference between SHU9119 (antagonist) and melanotan II (agonist) is located at a single position (red, D-Nal vs. D-Phe), leading to the opposite ligand effect (Nle: norleucine).

As already suggested in previous studies on ligand/MC4R contacts [25,63,181] and a recent study on the linear peptide α-MSH vs. the biased cyclic MSH derivative setmelanotide [39], a high number of intermolecular contacts between amino acid residues at the transmembrane region (TMHs 2–7) and ELs 2/3 are involved in α-MSH binding (Figure 7). The putative key interactions between agonistic ligands and MCRs are formed by the specific ligand core motif “HFRW” and different receptor residues, such as the receptor aspartates D122, D126, residues with aromatic ring systems (F261 and F284), H264, or hydrophobic amino acids, such as L265 and L288. This is likely common for agonistic MC4R ligand peptides. Specificities in the signaling profile, as observed for setmelanotide [39], in contrast to the linear MSH, can be attributed to the cyclic structure in comparison to the linear MSH peptide, and particular ligand differences in the amino acid composition and resulting receptor contacts. However, these predicted differences need to be deciphered more precisely under experimental conditions to also understand deeper causalities between specific ligand binding and biased signaling at the MCRs.

Moreover, the cyclic peptidic ligand SHU9119, which is bound in the crystal structure of the inactive state of the MC4R (Figure 4, [21]), reveals specific hydrogen bonds with receptor amino acids in TM2 (T101), TM3 (e.g., N123), EL2 (S188), and TM6 (H264). According to the crystal structure, these interactions are highly important for ligand formation and action. It will be interesting to examine the impact of these interactions for agonistic ligands and to finally discriminate between specific and common agonistic and antagonistic interactions at the molecular level. For example, the MC1R has a tyrosine instead of the MC4R serine 188 (Figure 7A). Serine 188 of MC4R forms a hydrogen bond network with the backbone amide of W7 and with the side chain to R6 of SHU9119 [21]. The much larger tyrosine of the MC1R likely does not interact with SHU9119 W7 as observed for the MC4R S188 and, remarkably, SHU9119 is a partial agonist on the MC1R [22,182]. Moreover, a previous report [183] identified residue L133 in TMH3 of MC4R (Figure 4B) as crucial for SHU9119 mediated effects. The single substitution of L133 to methionine did convert SHU9119 from an antagonist to an agonist. In contrast, substitution of M128 in the MC1R to leucine, the homologous residue 133 of hMC4R, displays a reduction in SHU9119 binding affinity and potency [183]. L133, together with L129 and I197 (TMH5), covers the bottom part of the SHU9119 binding pocket, and even the side chain of L133 is 3.6 Å in distance to the SHU9119 Nal side chain (Figure 7C). Detailed studies exploring how this L-M exchange transfers the antagonistic effect of SHU9119 to the opposite, together with observed interactions in EL2, will be of high pharmacological importance.

Of further note, the antagonist SHU9119 differs from the agonist melanotan II by a single amino acid, as depicted in Figure 7C. The D-Phe of melanotan II in the central core of the ligand is D-Nal (Naphtylalanine) in SHU9119. This difference leads to opposite functional effects. By using the new MC4R crystal structure and principle ligand (SHU9119) orientation as a template for α-MSH docking (Figure 7B), details of the putative MSH ligand binding site and participating receptor amino acids can be investigated. According to this model, one major difference between SHU9119 and α-MSH is the orientation of Trp in the central core motif, which is strongly dependent on the F7 (α-MSH) or the D-Nal (SHU9119), respectively. The tryptophan in α-MSH can enter the central region between the helices deeper towards W258 in TMH6, which is not feasible for the corresponding Trp in SHU9119 because the large and bulky D-Nal is a sterical barrier.

Although there are overlapping binding sites between agonist and the inverse agonist AgRP as evidenced by AgRP displacement of radiolabeled NDP-MSH in binding experiments, the contacts of AgRP to the MC4R are likely more widely distributed across different receptor parts, including distinct TMHs, the ELs, and the N-terminus [48]. Several supposed AgRP-specific interactions to the receptor (as, e.g., (*AgRP*/MC4R amino acid denotation): *F116*/F284, *Y109*/Y268, *Y118*/Y35, or *R120*/D189) may not be relevant for agonistic ligand binding. They help to explain the antagonistic and, especially, the inverse agonistic effect of AgRP, because these contacts should act as constraints, keeping the MC4R in an inactive conformation, at least for certain signaling pathways [113].

Concerning the antagonistic effect of AgRP, an alternative scenario was previously assumed [79], whereby the inverse agonist activity of AgRP on the MC4R should be attributed to inhibition of permanent (basal) activation mediated by the N-terminus. How this mechanism is analogous to the experimentally proven direct AgRP/MC4R interaction between the negatively charged receptor amino acid residues E100, D122, and/or D126 in the central part of the receptor TMD and R111 in the conserved and activity related AgRP _111_RFF_113_ motif [153,184,185,186] has not yet been clarified. The fact that calcium ions do not affect AgRP binding in contrast to other MC4R ligands [21] is of interest, clearly indicating differences between MC4R ligands and the relationship to ion binding.

**Figure 8:**
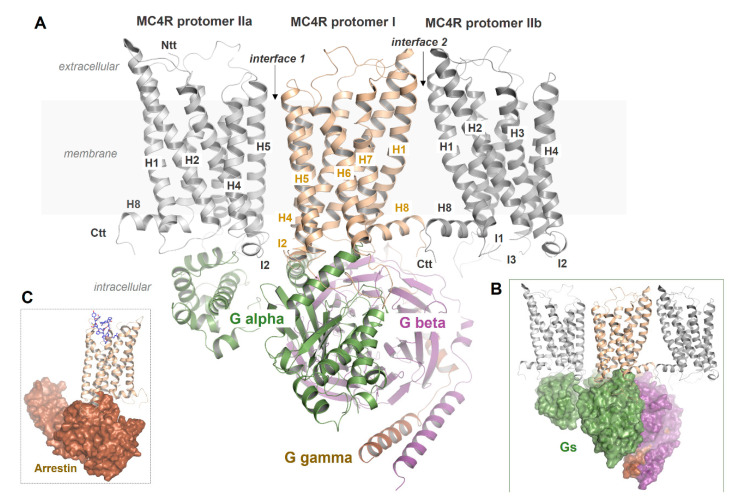
Putative MC4R dimer or oligomer constellations with bound G protein. **A**: The MC4R can represent dimers at the IL2–TMH4 interface; however, other interfaces, such as the TMH1–helix 8 interface, cannot be excluded for oligomers. In the supposed arrangement of an interface between IL2 and TMH4, for steric reasons, only one G protein molecule can likely bind (**B**). It remains unknown how MC4R oligomerization affects β-arrestin binding (**C**) or how receptor protomers in oligomers differ from each other in their ligand binding kinetics through mutual influence, or how they react to simultaneously available arrestin and G protein molecules.

Generally, diverse ligand binding, as described above, and subsequent manifold modulated signals at the MC4R are very complex and additionally tuned by several additional external factors. First, a novel endogenous agonistic ligand, lipocalin-2, was recently discovered to interact with the MC4R [45]. Second, there are specific binding kinetics in oligomeric MC4Rs [187], whereby the interacting monomer/ligand complexes may exhibit mutual cooperativity and cross-interactions, which have only been investigated incompletely to date [91]. Third, the co-expression of the MC4R interacting accessory protein MRAP2 leads to an alteration in the specificity of ligand binding at the MC4R, therefore resulting in high MC4R sensitivity for ACTH [44], which usually primarily activates the MC2R. The direct and physiologically relevant MRAP2–MC4R interaction should be of high importance for all studies both in vivo and in vitro, since, for example, in such heteromeric constellations, the postulated MC4R ion binding capacities (Figure 6) or receptor–receptor oligomeric states (Figure 8) could be strongly modified, as is the case for the above-mentioned class B GPCRs interacting with RAMPs. The relevance of MRAP2 on diverse GPCRs as a regulatory element has also been shown for other class A GPCRs, such as the ghrelin receptor (GHSR) [188], the orexin receptor 1 (OX1R) [189], or the prokineticin receptor-1 (PKR1) [190], clearly indicating a more extended physiological impact of MRAPs on class A GPCRs [42].

### 2.7. MC4R Assembly in Multimeric Constellations

Homo- [191] and hetero-oligomerization are widely accepted to be highly significant for GPCR functionalities [192]. The ability to form multimeric receptor assemblies affects physiological aspects [193,194,195,196] but is also associated with pathophysiological/pharmacological issues [197,198,199,200,201]. GPCR oligomerization can lead to modified ligand binding [187,202,203,204], G protein selectivity [205], signaling [206,207,208], or cell surface expression [209,210] and internalization [211]. Allosteric or cooperative effects between individual protomers in oligomeric receptor constellations have been observed [212,213]. GPCR constellations could be a mixture of monomers and oligomers that are dynamically interconverted among the various forms [214], suggesting that GPCRs exist in a monomer/oligomer ratio at the membrane, presumably in different cases also in intracellular compartments [215,216,217].

The MC4R is also known to form homo-oligomers [40,41,89] as well as heteromers with GPR7 [218] or MC3R [59]. Further support on MC4R oligomerization is provided by studies on ligand binding kinetics, whereby two different ligand binding sites (in two interacting protomers) in oligomeric MC4R complexes are presumably interrelated [187]. Moreover, the two moieties of a (homo- or hetero-) bivalent MC4R ligand [57,58,59,60,219] may bind each at a single MC4R protomer in a dimeric receptor constellation, as shown for bivalent ligands at the oxytocin receptor dimer [220].

Unfortunately, studies on the precise formation of MC4R oligomers are almost lacking. In general, based on previously determined crystal structures of class A GPCR oligomers and biophysical studies, several oligomeric GPCR–protomer interfaces in oligomers have been proposed [221] or predicted ab initio [222,223]. For example, in opsin [224,225,226,227], κ-opioid receptor (KOR [228]), and β1-adrenergic receptor 1 (β1-AR, [229]), the protomer interface is located at TMH1–TMH2 and helix 8. Oligomeric contacts are also known between GPCR protomers at the region ICL2/TMH4 [230,231,232], between TMH4 and TMH5 [233], or are located at TMH5–TMH6 [234,235,236]. The new MC4R crystal structure (PDB ID: 6W25), which is in an inactive state, was crystallized as an artificial dimer in the crystal packing with a head-to-tail monomer–monomer interface and, therefore, no relevant information for homo-dimerization can be derived [21]. In conclusion, many different protomer–protomer interfaces can be assumed for GPCRs, independent of functional relevance. The simultaneous occurrence of more than one interface in a particular receptor oligomer is rather likely. The importance of MC4R oligomerization was recently highlighted by a study showing transactivation between MC4R protomers in a dimer, suggesting functionally relevant crosstalk between the receptor subunits in the oligomeric complex [91].

In accordance with this, a previous report on MC4R showed that specific modifications in a region between TMH3 and IL2 and TMH4 led to forced oligomer separation, presumably into receptor monomers [90]. Not only does this indicate a distinct interface between MC4R protomers located in this structural part (Figure 8), but surprisingly also the maximum signaling capacity (Gs activation) for these monomerized receptor constructs was approximately doubled. This may point to differences between the signaling properties of MC4R monomers vs. oligomers with regard to MC4R/G protein stoichiometry, as discussed for other GPCRs [237]. In specific GPCR dimeric constellations, only a single G protein molecule can access two activated protomers for sterical reasons (stoichiometry 2:1) (Figure 8), while monomerized receptors each couple to one G protein molecule (stoichiometry 2:2), which may lead to a doubling of signaling capacity in terms of G protein activation. In turn, oligomerization and/or oligomer separation should also be fine-tuning elements to control signaling levels or selectivity at the intracellular region.

## 3. Concluding Remarks

The MC4R is a promising target for drugs to treat obesity or sexual dysfunction. An immense amount of information on the prevalence and dysfunctions of over 100 pathogenic MC4R mutations is available, as well as pharmacological profiles and effects of already designed MC4R ligands. The recently published inactive MC4R crystal structure with a bound peptidic antagonist and a Ca^2+^ ion [21] is an important step forward in understanding this receptor at the atomic level. However, in view of the well-known MC4R features regarding the importance of biased signaling, basal signaling, signal modulation by interacting partners, such as MRAP2 or oligomerization, it will be of enormous importance to gain further structural insights into the active receptor state in complex with agonists or with signal effectors such as G proteins or arrestins. This is highly relevant for optimizing known MC4R ligands towards increased selectivity to certain signaling pathways, such as Gs or Gq, but also to make the ligands more specific for individual MCR subtypes. In the future, MC4R research should also focus on optimizing MC4R ligands that induce or avoid homo- and hetero-oligomerization to receive higher selectivity. Finally, the information reviewed here based on currently published knowledge points to a very complex system of factors involved in the physiologically relevant regulation of the MC4R and homologous GPCRs. It will be a future challenge to assemble all factors and participating proteins into a unifying and comprehensive concept of MC4R activity regulation.
